# Tourniquet Use and Local Tissue Concentrations of Cefazolin During Total Knee Arthroplasty: A Randomized Clinical Trial

**DOI:** 10.1001/jamanetworkopen.2024.29702

**Published:** 2024-08-23

**Authors:** Julien Montreuil, Michael Tanzer, Yu Ling Zhang, Ewa Rajda, Daina Avizonis, Adam Hart

**Affiliations:** 1 Division of Orthopaedic Surgery, McGill University, Montreal, Quebec, Canada; 2 Jo Miller Orthopaedic Research Laboratory, Montreal, Quebec, Canada; 3 Research Institute, McGill University Health Centre, Montreal, Quebec, Canada; 4 Division of Infectious Diseases, McGill University, Montreal, Quebec, Canada; 5 Metabolomics Innovation Resource, Rosalind and Morris Goodman Cancer Institute, McGill University, Montreal, Quebec, Canada

## Abstract

**Importance:**

Prophylactic administration of antibiotics before skin incision is an important component in the prevention of periprosthetic joint infection in arthroplasty surgery. For antibiotics to be effective, the local tissue concentration (LTC) must exceed the minimum inhibitory concentration of typical infecting organisms; however, the LTC of cefazolin during arthroplasty is poorly understood.

**Objective:**

To compare the systemic concentration of cefazolin in serum with the LTC in fat, synovium, and bone during primary total knee arthroplasty (TKA) while assessing the effect of tourniquet inflation.

**Design, Setting, and Participants:**

This prospective randomized clinical trial was conducted from March 1, 2022, to June 30, 2023, in patients undergoing TKA at a single academic center.

**Intervention:**

Total knee arthroplasty with or without a limb tourniquet.

**Main Outcomes and Measures:**

Systemic blood and local tissues from the surgical site (fat, synovium, and bone) were harvested at regular intervals during the surgery. The primary outcome was the LTC of cefazolin, quantified using the liquid chromatography–tandem mass spectrometry technique.

**Results:**

A total of 59 patients were included in the study, with 29 in the tourniquet group (mean [SD] age, 69.3 [9.6] years; 23 [79.3%] female) and 30 in the no tourniquet group (mean [SD] age, 69.9 [9.7] years; 21 [70.0%] female). In patients undergoing TKA without a tourniquet, the mean concentration of cefazolin in serum was 71.9 μg/mL (95% CI, 66.4-77.5 μg/mL), whereas the mean LTCs were 13.9 μg/g (95% CI, 12.1-15.7 μg/g) in fat, 27.7 μg/g (95% CI, 24.3-31.0 μg/g) in synovium, and 17.7 μg/g (95% CI, 14.8-20.5 μg/g) in bone. For patients undergoing TKA with a tourniquet, the mean concentration of cefazolin in serum was 72.0 μg/mL (95% CI, 66.3-77.7 μg/mL), and the mean LTCs were 9.9 μg/g (95% CI, 8.7-11.1 μg/g) in fat, 21.8 μg/g (95% CI, 18.7-25.0 μg/g) in synovium, and 13.0 μg/g (95% CI, 10.8-15.2 μg/g) in bone. The use of a tourniquet resulted in significantly lower mean LTCs by 60 minutes after cefazolin infusion (10.8 μg/g [95% CI, 9.1-12.4 μg/g] vs 16.9 μg/g [95% CI, 14.1-19.6 μg/g], *P* = .001 in fat; 18.9 μg/g [95% CI, 14.1-23.6 μg/g] vs 25.8 μg/g [95% CI, 21.4-30.3 μg/g], *P* = .03 in synovium; and 11.8 μg/g [95% CI, 9.3-14.2 μg/g] vs 19.4 μg/g [95% CI, 14.5-24.4 μg/g], *P* = .007 in bone).

**Conclusions and Relevance:**

In this randomized clinical trial, the concentration of cefazolin was lower in local tissues (fat, synovium, and bone) than in systemic blood, and the use of a limb tourniquet further significantly reduced these concentrations. Although the current prophylactic dosing regimen for cefazolin provides sufficient serum concentrations, the levels in the periarticular tissue during TKA may be insufficient to prevent periprosthetic joint infection.

**Trial Registration:**

ClinicalTrials.gov Identifier: NCT05604157

## Key Points

**Question:**

What is the effect of tourniquet application on local tissue concentration of cefazolin during total knee arthroplasty?

**Findings:**

In this randomized clinical trial of 59 adults, the use of a tourniquet resulted in significantly lower concentrations in fat, synovium, and bone by 60 minutes after cefazolin infusion. The mean concentration of cefazolin measured in the local tissues ranged from 9.9 to 21.8 μg/g for the tourniquet group and was insufficient to adequately cover pathogens with elevated minimum inhibitory concentration.

**Meaning:**

The study underscores the adverse effect of tourniquet inflation on tissue concentration of antibiotics and raises questions regarding cefazolin’s effectiveness against pathogens with higher minimum inhibitory concentrations.

## Teaser

This randomized clinical trial of patients undergoing total knee arthroplasty examines the effect of tourniquet application during the procedure on local tissue concentrations of cefazolin.

## Introduction

Surgical site infections are now the most common and costly of all hospital-acquired infections, accounting for 20% of all nosocomial infections. These infections are associated with a 2- to 11-fold increase in the risk of mortality.^1^ Periprosthetic joint infections (PJIs) are the most common cause of early revision surgery after total joint arthroplasty, with up to 55% occurring within 90 days after implantation and 34% within 2 years.^2^ The projected annual hospital costs of PJI in the US for total joint arthroplasty are estimated to be $1.85 billion by 2030.^3,4^ Periprosthetic joint infections occurring soon after surgery are often acquired during implantation; therefore, prevention at the time of surgery will have the greatest impact.^5^

Prophylactic administration of intravenous (IV) antibiotics before skin incision is a crucial component in the prevention of PJI in arthroplasty surgery alongside other important measures, such as surgical technique, aseptic protocols, and patient optimization.^6^ A first-generation cephalosporin, cefazolin, is the recommended antibiotic of choice in arthroplasty procedures because of its broad-spectrum activity against *Staphylococcus* species and *Streptococcus* species, its tissue penetration, minimal toxicity, and relatively low cost.^3,7^ Although level 1 evidence demonstrates a significant reduction in surgical PJI with a preincisional dose of a first-generation cephalosporin, further details on the exact timing, correlation to length of surgery, effect of weight, and influence of a limb tourniquet are not well defined.^8,9^ Because medications are not equally distributed throughout the body, it is critical to know that an antibiotic obtains adequate concentrations in serum as well as the local tissues at the surgical site. For the antibiotic to be effective, the local tissue concentration (LTC) must exceed the minimum inhibitory concentration (MIC) of infecting organisms for the duration of the surgery.^10,11^ The MIC of cefazolin varies depending on the species of the bacteria and antibiotic resistance mechanisms. *Staphylococcus aureus* and coagulase-negative staphylococci are 2 of the most common pathogens naturally found on the skin and are responsible for approximately half of PJIs. Although the MIC itself does not vary among different tissues, the distribution and concentration of the drug in various matrices, such as blood, fat, and bone, can differ due to pharmacokinetic properties. Although the MIC of *S aureus* ranges from 0.5 to 8 μg/mL,^12,13,14^ many coagulase-negative staphylococci have MICs ranging from 2 to 50 μg/mL and are reported as an increasingly common causal agent of PJI.^11^

Total knee arthroplasty (TKA) is still commonly performed with or without a limb tourniquet, with benefits and harms intensely debated during the past decade.^15^ A tourniquet can be used to apply high pressure around the thigh to reduce blood flow and improve bone cementing.^15^ A meta-analysis recently reported that the use of tourniquets in TKA is associated with an increased risk of infection.^16^ This finding underscores the importance of investigating the LTC of antibiotics as an objective measure to understand the mechanisms by which tourniquet use may negatively impact surgical site infection rates. The aim of this randomized clinical trial was to examine the concentration of cefazolin in systemic blood as well as LTCs in fat, synovium, and bone during TKA while assessing the effect of tourniquet inflation.

## Methods

### Population

This prospective, single-center, intention-to-treat randomized clinical trial studied patients undergoing TKA at a tertiary care academic hospital. Patients undergoing revision surgery, those with severe allergy to cefazolin, those with severe kidney dysfunction, those who weighed 120 kg or more, and those with methicillin-resistant *S aureus* colonization were excluded from the study. Race reporting was omitted because local antibiotic concentrations are primarily influenced by individual physiologic factors rather than racial characteristics. The trial was retrospectively registered due to operational delays caused by the COVID-19 pandemic despite obtaining local institutional review board approval (McGill University Health Centre Authorization [2021-6782]) well before recruitment. Sixty-one patients were recruited and provided written informed consent. Patients were randomized (1:1) to the tourniquet group (TG) or no tourniquet group (NTG) from March 1, 2022, to June 30, 2023, using computer-generated random allocations in numbered opaque, sealed envelopes. Of the 61 recruited and randomized patients, 2 were excluded due to the inability to obtain a dedicated IV access for intraoperative blood sampling. This report follows the Consolidated Standards of Reporting Trials (CONSORT) reporting guideline. The trial protocol can be found in Supplement 1.

**Figure 1.**
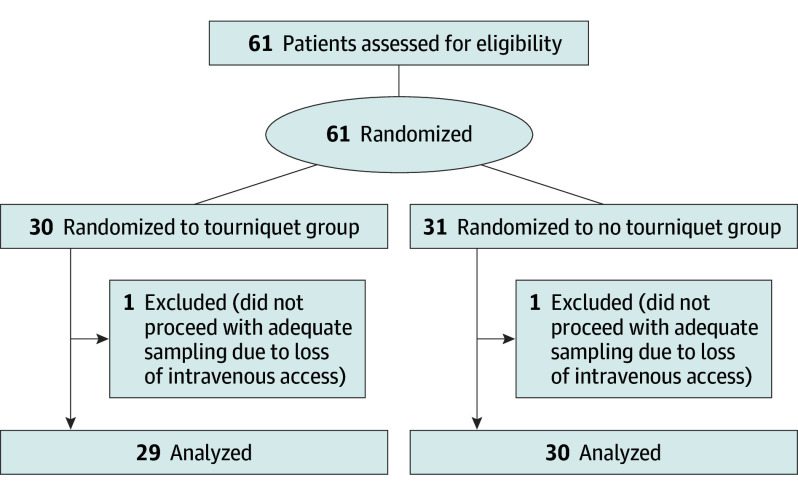
Flow Diagram

The primary outcome was to evaluate the LTC of cefazolin with or without the use of a limb tourniquet during surgery. The secondary outcomes were the effects of time from infusion to incision, patient weight, and length of surgery on the LTC of cefazolin. The 25 participants per group specified at registration were intended as a minimum threshold, and the additional patients were recruited to ensure robustness and reliability of our results. On enrollment, comprehensive demographic and perioperative temporal metrics were collected to facilitate a thorough analysis of the secondary outcomes (Figure 1).

### Sample Collection and Timing

Patients were given 2 g of cefazolin as an IV bolus within 1 hour of the incision. The infusion was completed at least 5 minutes before the tourniquet inflation (TG) or the skin incision (NTG). The time of IV infusion was noted, and a running timer was started from that point. A limb tourniquet (Zimmer Biomet) was inflated to a pressure of 250 mm Hg for patients in the TG. The time of tourniquet inflation and deflation as well as the skin incision was noted. Blood samples, to determine systemic blood levels of cefazolin, were taken from a dedicated IV catheter in the patient’s arm away from any other IV accesses being used to administer fluids or medications. Blood samples were placed in EDTA tubes. Local tissue samples of fat, synovium, and bone measuring at least 0.5 cm^2^ were harvested using sharp excision. Bone and synovium samples were obtained from the distal femur. Samples were placed into sterile containers and immediately stored at −80 °C. The first samples of blood and fat were obtained immediately at the time of the skin incision (Figure 1). An alarm rang every 30 minutes from the time of cefazolin administration on a recurring basis to obtain the subsequent samples (systemic blood and local fat, synovium, and bone) at T1, T2, and T3, corresponding to 30, 60, and 90 minutes after cefazolin administration, respectively. The time of the final TKA insertion was recorded. Finally, a blood sample was obtained at least 1 hour after the surgery in the recovery room with time noted. The number of samples collected for each patient varied due to the individual surgical times. A longer surgery duration typically allowed for the collection of more samples from various tissues.

### Liquid Chromatography–Tandem Mass Spectrometry Technique

Serum and LTC quantification of cefazolin in fat, synovium, and bone tissues was performed using our previously validated and published liquid chromatography–tandem mass spectrometry (LC-MS) method.^17^ This protocol was rigorously tested, including measurements of reproducibility and calibration curve quality. The recovery of the extraction method ranges from 94% to 113% across all sample types.^17,18^ Cefazolin levels were detected using ultrahigh-pressure chromatography (1290 UPLC, Agilent Technologies) coupled to a triple quadrupole mass spectrometer (6470 QQQ, Agilent Technologies). All samples were analyzed in triplicate to ensure precision.

### Statistical Analysis

Means, SDs, and 95% CIs were calculated for the cefazolin concentrations in the different samples. A 2-tailed, unpaired *t* test and analysis of variance were used for repeated measures of cefazolin concentration between groups. A minimal sample size of 50 patients was calculated for a double-sided test with an α = .05, power of 90%, estimated clinically significant difference of 25% (5 μg/g) between groups, and an expected SD of 5 μg/g in the tissue concentration measurement based on previous studies.^11,12,19^ Similar to other comparative studies, LTCs are compared with standard MIC as surrogate given tissue densities of 1 g/mL.^11,12^ A 2-sided *P* < .05 was considered to be statistically significant.

## Results

**Figure 2.**
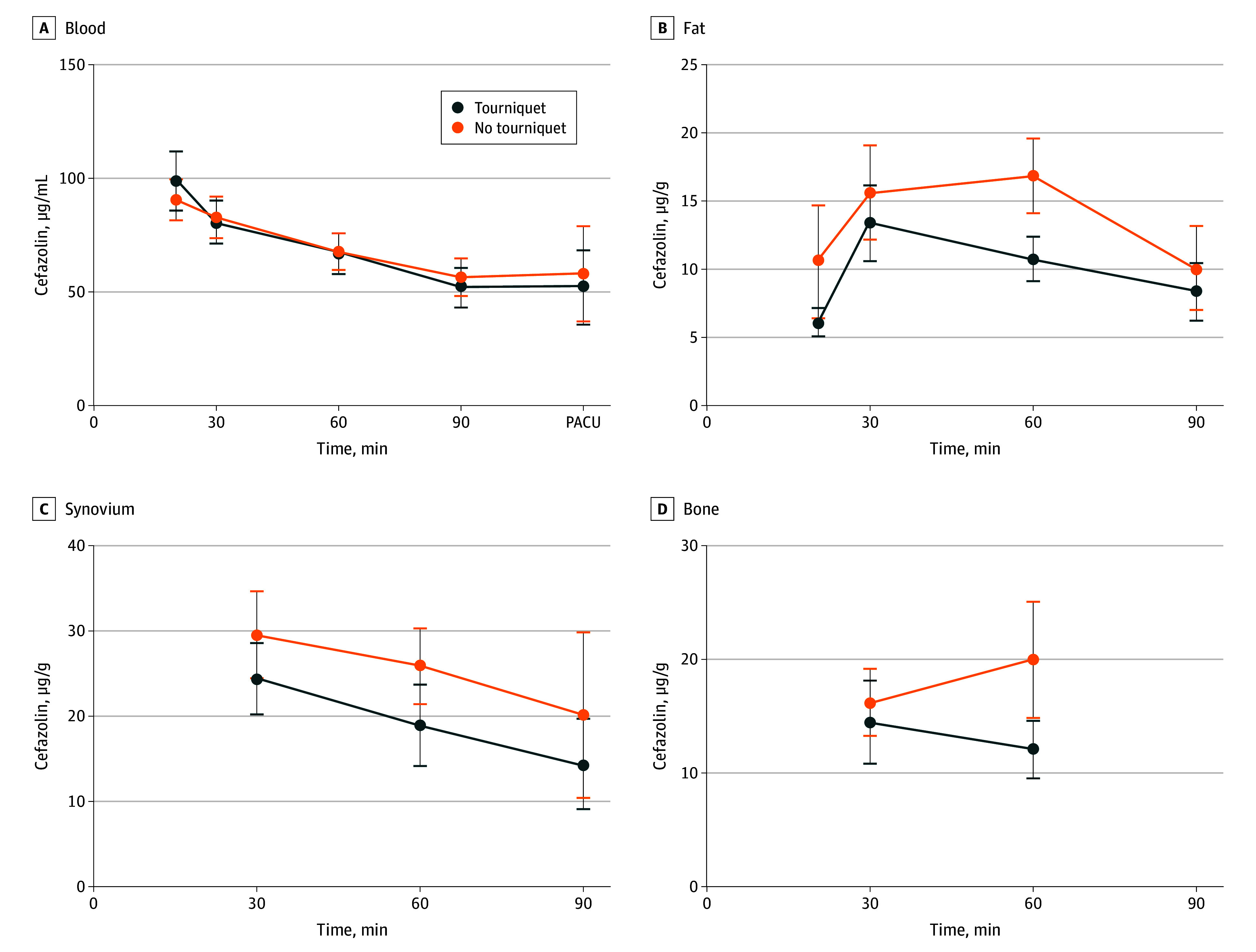
Cefazolin Concentration in Blood, Fat, Synovium, and Bone With and Without Tourniquet Error bars indicate 95% CIs. PACU indicates postanesthesia care unit.

A total of 59 patients were analyzed, including 29 in the TG and 30 in the NTG. No substantial differences were found in the baseline characteristics between the 2 groups, including sex, age, and body mass index (BMI; calculated as weight in kilograms divided by height in meters squared) (Table 1). In the TG, 6 patients (20.7%) were male and 23 (79.3%) were female, with a mean (SD) age of 69.3 (9.6) years and a mean (SD) BMI of 29.3 (5.3). Conversely, in the NTG, 9 patients (30.0%) were male and 21 (70.0%) were female, with a similar mean (SD) age of 69.9 (9.7 years) and a mean (SD) BMI of 30.1 (5.5). All patients received 2 g of cefazolin within 5 to 60 minutes of incision. The mean (SD) time from cefazolin infusion to incision was 26.2 (10.6) minutes for the TG and 24.1 (9.5) minutes for the NTG (*P* = .40). The mean (SD) surgical time was 77.8 (16.7) minutes for the TG and 81.4 (19.0) minutes for the NTG (*P* = .40). The mean (SD) tourniquet time was 57.0 (19.6) minutes (Figure 2 and Table 1).

**Table 1.** Demographics and Baseline Characteristics

Characteristic	Mean (SD)	
	Tourniquet (n = 29)	No tourniquet (n = 30)
Sex, No. (%)		
Male	6 (20.7)	9 (30.0)
Female	23 (79.3)	21 (70.0)
Age, y	69.3 (9.6)	69.9 (9.7)
BMI	29.3 (5.3)	30.1 (5.5)
TTI, min	26.2 (10.6)	24.1 (9.5)
Surgical time, min	77.8 (16.7)	81.4 (19.0)
Tourniquet time, min	57.0 (19.6)	NA
Time from cefazolin to deflation, min	85.0 (22.0)	NA
Time from cefazolin to PACU, min	202.2 (65.9)	194.2 (68.9)

Abbreviations: BMI, body mass index (calculated as weight in kilograms divided by height in meters squared); NA, not applicable; PACU, postanesthesia care unit; TTI, time to incision.

The mean concentration of cefazolin in serum in the TG was 72.0 μg/mL (95% CI, 66.3-77.7 μg/mL), whereas the mean LTCs of cefazolin were 9.9 μg/g (95% CI, 8.7-11.1 μg/g) in fat, 21.8 μg/g (95% CI, 18.7-25.0 μg/g) in synovium, and 13.0 μg/g (95% CI, 10.8-15.2 μg/g) in bone. For the NTG, the mean concentration of cefazolin in serum was 71.9 μg/mL (95% CI, 66.4-77.5 μg/mL), whereas the mean LTCs of cefazolin were 13.9 μg/g (95% CI, 12.1-15.7 μg/g) in fat, 27.7 μg/g (95% CI, 24.3-31.0 μg/g) in synovium, and 17.7 μg/g (95% CI, 14.8-20.5 μg/g) in bone. Overall, the TG had significantly lower mean (SD) LTCs in all 3 tissues compared with the NTG (fat: *P* = .001; synovium: *P* = .01; bone: *P* = .01) (Table 2).

**Table 2.** Cefazolin LTCs in All Tissues at Different Time Points^a^

Tissue	Tourniquet			No tourniquet			*P* value
	LTC, mean (SD)	95% CI	No. of samples	LTC, mean (SD)	95% CI	No. of samples	
**Incision** ^b^							
Blood	99.7 (33.0)	86.0-112.1	27	90.9 (22.7)	81.9-99.8	27	.29
Fat	6.1 (2.7)	5.0-7.2	26	10.5 (9.9)	6.3-14.7	25	.03
**T1 (30 min after infusion)**							
Blood	81.2 (25.2)	71.7-90.8	29	83.1 (24.6)	74.0-92.4	30	.77
Fat	13.4 (7.3)	10.6-16.1	29	15.7 (8.9)	12.2-19.1	28	.28
Synovium	24.4 (10.8)	20.2-28.6	28	29.5 (13.4)	24.4-34.6	29	.12
Bone	14.1 (9.0)	10.5-17.6	27	15.8 (6.8)	12.9-18.7	24	.45
**T2 (60 min after infusion)**							
Blood	67.1 (23.1)	58.2-76.1	28	68.0 (19.9)	60.0-76.1	26	.88
Fat	10.8 (4.0)	9.1-12.4	25	16.9 (7.1)	14.1-19.6	27	.001
Synovium	18.9 (11.3)	14.1-23.6	25	25.8 (11.7)	21.4-30.3	28	.03
Bone	11.8 (5.7)	9.3-14.2	24	19.4 (12.1)	14.5-24.4	24	.007
**T3 (90 min after infusion)**							
Blood	52.2 (20.0)	43.6-60.9	23	56.8 (20.1)	48.7-65.0	24	.43
Fat	8.4 (3.3)	6.2-10.4	12	10.1 (5.1)	7.0-13.2	12	.33
Synovium	14.4 (4.3)	9.1-19.6	5	20.1 (12.7)	10.4-29.8	8	.35
Bone	NA	NA	NA	NA	NA	NA	NA
**Recovery room** ^c^							
Blood	52.4 (35.6)	36.2-68.6	21	58.5 (52.1)	37.5-79.5	26	.65
**Global mean**							
Blood	72.0 (32.6)	66.3-77.7	128	71.9 (32.7)	66.4-77.5	133	.90
Fat	9.9 (5.7)	8.7-11.1	92	13.9 (8.6)	12.1-15.7	92	.001
Synovium	21.8 (11.3)	18.7-25.0	58	27.7 (12.6)	24.3-31.0	65	.01
Bone	13.0 (7.7)	10.8-15.2	51	17.7 (9.9)	14.8-20.5	48	.01

Abbreviations: LTC, local tissue concentration; NA, not applicable.

^a^ For LTCs, blood was measured in micrograms per liter, and fat, synovium, and bone were measured in micrograms per gram.

^b^ Mean (SD) time to incision was 26.2 (10.6) minutes in the tourniquet group and 24.1 (9.5) minutes in the no tourniquet group.

^c^ Mean (SD) time to recovery was 202.2 (65.9) minutes in the tourniquet group and 194.2 (68.9) minutes in the no tourniquet group.

At 60 minutes after the cefazolin infusion, the mean LTCs were significantly lower in the TG than the NTG (10.8 μg/g [95% CI, 9.1-12.4 μg/g] vs 16.9 μg/g [95% CI, 14.1-19.6 μg/g], *P* = .001 in fat; 18.9 μg/g [95% CI, 14.1-23.6 μg/g] vs 25.8 μg/g [95% CI, 21.4-30.3 μg/g], *P* = .03 in synovium; and 11.8 μg/g [95% CI, 9.3-14.2 μg/g] vs 19.4 μg/g [95% CI, 14.5-24.4 μg/g], *P* = .007 in bone). Both serum and tissue concentrations of cefazolin decreased over time. At the T2 point, a decrease in LTC levels was observed in both fat and synovium compared with the initial levels at T1 with the exception of the fat samples in the NTG, which maintained a relatively stable concentration level similar to T1. These decreasing concentrations persisted, and by T3, all tissue concentration levels had continued to decrease. At the postoperative time point in the recovery room, the serum level of cefazolin was 72% of the incisional level for the TG and 79% for the NTG at 202.2 and 194.2 minutes, respectively, since antibiotic administration (Table 2).

Overall, there were no differences in LTCs based on the timing of cefazolin infusion before the skin incision. Operations that commenced less than 20 minutes after cefazolin infusion exhibited LTCs comparable to those between 20 and 40 minutes or those between 40 and 60 minutes (Table 3 and eTable in Supplement 2). Individuals classified as having obesity (BMI ≥30) had reduced LTCs in the sampled fat tissue when compared with their counterparts without obesity at both T1 and T2.

**Table 3.** Effect of TTI on LTC at Incision and 30 Minutes After Cefazolin Administration^a^

Time point	TTI <20 min			TTI 20 to <40 min				TTI ≥40 min			*P* value
	LTC, mean (SD)	95% CI	No. of samples	LTC, mean (SD)	95% CI	No. of samples	LTC, mean (SD)		95% CI	No. of samples	
Incision											
Blood	95.7 (20.7)	84.7-106.8	16	98.6 (31.7)	86.9-110.2	31	77.1 (23.3)		55.5-98.7	7	.19
Fat	6.3 (3.6)	4.3-8.4	14	9.7 (8.9)	6.4-13.1	30	5.2 (1.0)		4.3-6.2	7	.19
T1 (30 min)											
Fat	13.2 (7.7)	9.2-17.1	17	15.3 (8.8)	12.2-18.3	34	13.9 (5.7)		7.9-19.9	6	.68
Synovium	24.2 (11.1)	18.3-30.1	16	28.3 (12.7)	23.9-32.7	35	26.7 (14.4)		11.6-41.8	6	.56
Bone	13.6 (6.9)	9.4-17.7	13	15.9 (8.9)	12.8-19.1	32	12.2 (4.7)		7.3-17.0	6	.46

Abbreviations: LTC, local tissue concentration; TTI, time to incision.

^a^ For LTCs, blood was measured in micrograms per liter, and fat, synovium, and bone were measured in micrograms per gram.

## Discussion

Although prophylactic antibiotics are well established to reduce PJI, the effect of a tourniquet on the LTC of cefazolin in fat, synovium, and bone is not known. Furthermore, the LTC of cefazolin during TKA and the influence of BMI as well as time from cefazolin infusion to incision on LTC are not known. In this randomized clinical trial, the LTC of cefazolin was lower in fat, synovium, and bone in the TG compared with the NTG. The difference was significant in all tissues at more than 60 minutes after cefazolin administration.

The disparity in the concentration levels of cefazolin in serum vs the local tissues is striking. Although the blood maintains a cefazolin concentration well above the desired MIC of the most common infecting pathogens for the duration of the surgery, the LTCs of cefazolin in fat, synovium, and bone marrow are notably lower. The mean tissue concentration levels observed in our study, falling within the range of 10 to 20 μg/g for the tourniquet group, are likely sufficient to combat some pathogens, such as methicillin-susceptible *S aureus*, but insufficient for other common infecting pathogens, such as coagulase-negative staphylococci. Our findings suggest that the current guidelines of cefazolin use for arthroplasty surgery may be insufficient. Maintaining the LTC above the MIC is crucial to prevent PJI, minimize the development of antibiotic-resistant bacteria, and enhance surgical outcomes.

The use of a tourniquet during TKA further reduced the LTCs of cefazolin. Although the benefits and harms of tourniquet use are intensely debated in the arthroplasty literature, the findings from this study raise concerns for its use in accordance with a recent meta-analysis that found that the use of tourniquets in TKA is associated with an increased risk of infection.^16^ The LTC of antibiotics is one of many objective measures for studying the mechanisms by which tourniquet use may negatively impact surgical site infection rates. Our study suggests that reducing circulation to the leg may hinder the distribution of antibiotics to peri-incisional tissues, potentially compromising the effectiveness of cefazolin prophylaxis during tourniquet inflation. Further investigation is necessary to assess whether adjustments to surgical techniques or dosing regimens should be considered. For instance, Young et al^12^ have already shared findings that indicate the significant effect of intraosseous administration of antibiotics in increasing LTCs. Another recommendation from our study would be to limit tourniquet inflation solely to the cementation of implants during the procedure. The effect of a temporary decrease in the LTC of cefazolin during inflation and PJI remains unknown.

The blood concentration of cefazolin remained above MICs for most common pathogens throughout the surgery but decreased over time. This finding is consistent with the known pharmacokinetics of cefazolin, which exhibits a rapid initial distribution phase followed by a slower elimination phase. Although the decreasing blood concentrations are expected, it is important to note that the antibiotic’s effect on local tissues persists due to its ability to penetrate and accumulate in various tissues. We hypothesized that application of a tourniquet would affect the peak tissue concentration of cefazolin while prolonging its elimination. In fact, in this study, regardless of tourniquet application, LTC in both fat and synovium seemed to plateau and decrease after 60 minutes. Although current Centers for Disease Control and Prevention guidelines only advocate for a preincision dose of cefazolin with redosing at 4-hour intervals,^3^ our findings suggest that redosing sooner could be necessary to maintain appropriate MIC in the local tissues. A similar study conducted during revision arthroplasty, which typically has a longer duration, would likely provide a better assessment of the time-dependent efficacy of cefazolin and highlight the importance of redosing to maintain optimal antibiotic levels throughout the procedure. Such a study could ensure better prophylaxis against infections, especially for longer operations. Adequate and sustained antibiotic concentrations in tissues and serum are crucial to prevent infection at the surgical site.

Additionally, further analysis revealed that obesity led to lower LTCs in fat. This finding suggests that BMI may hold greater relevance than body weight alone when determining the appropriate antibiotic dose. Hussain et al^20^ shared that the dosing recommendations in weight-based dosing guidelines were primarily derived from small-scale and inconsistent pharmacokinetic studies. Body mass index accounts for both weight and height, providing a more comprehensive assessment of an individual’s body composition. Using BMI as a guide for antibiotic dosing could help ensure that the drug is administered at levels sufficient to achieve optimal therapeutic outcomes while minimizing potential adverse effects. These findings highlight the need for further research to elucidate the underlying mechanisms responsible for this effect.

This study challenges the conventional belief that a precise interval between antibiotic infusion and skin incision is a determinant in achieving adequate LTC. This study did not reveal any significant differences in LTC between patients with a time from cefazolin infusion to incision below or above 20 minutes. The findings of Hanberg et al^21^ align with the observation that minimal time is needed after the antibiotic bolus to achieve adequate concentration in tissues. There are notable differences between their study and our study. Specifically, their sample collection and microdialysis method differ significantly from our extraction technique. These methodologic disparities may contribute to variations in the observed tissue concentrations and tourniquet effect. Nonetheless, clinicians should continue to adhere to established guidelines, but they should also be aware that a rigid adherence to a specific time from cefazolin infusion to incision may not be the sole factor influencing LTC levels.

### Limitations

Our study has limitations that should be considered when interpreting the results. One of the primary limitations is that we measured total cefazolin LTC without considering the proportion of active cefazolin (unbound) in the tissues. Cefazolin’s efficacy is largely dependent on the concentration of unbound (free) antibiotic because it is the active form responsible for inhibiting bacterial growth. Although we acknowledge the variability of protein binding in serum and plasma as reported by Jongmans et al,^22^ the binding of cefazolin in tissues and measurement techniques are not well described in the current literature. Applying an arbitrary multiplier may not significantly alter the conclusions drawn from our study. The bound fraction of cefazolin might not contribute to the antimicrobial effect and could potentially lead to an overestimation of the actual antibiotic activity in the tissues. Furthermore, the study’s scope was limited to cefazolin, and other antibiotics that are less commonly used for prophylaxis in arthroplasty operations were not investigated. Different antibiotics may exhibit varying pharmacokinetic profiles and tissue penetration capabilities, which could lead to different LTC patterns in surgical tissues. Finally, our study did not explore the clinical outcomes and occurrence of PJI. Although measuring LTC is crucial to understand antibiotic distribution, the relationship between LTC and surgical site infection rates or patient outcomes warrants future investigation.

## Conclusions

This randomized clinical trial, using a precise LC-MS method, provides valuable insights into cefazolin’s LTCs during TKA. The findings in this study raise questions regarding cefazolin’s effectiveness against pathogens with higher MICs, such as coagulase-negative staphylococci. The study underscores the adverse effect of tourniquet inflation on LTC and the influence of obesity on antibiotic distribution. Although the current prophylactic dosing regimen for cefazolin provides sufficient serum concentrations, the concentrations in the periarticular tissue during TKA may be inadequate to provide the MIC necessary for the duration of the surgery to be effective in preventing PJI. Further research is required to further enhance our understanding of antibiotic concentration dynamics during surgery and to optimize prophylactic antibiotic protocols.

## Notes

Supplement 1.
Trial Protocol

Supplement 2. **eTable.** Effect of Obesity in Fat LTC at All Time Points

Supplement 3.
Data Sharing Statement
