# Supplementary material for: Tourniquet Use and Local Tissue Concentrations of Cefazolin During Total Knee Arthroplasty: A Randomized Clinical Trial
Source: JAMA Netw Open. 2024 Aug 23;7(8):e2429702. doi: 10.1001/jamanetworkopen.2024.29702 (PMC11344230; doi:10.1001/jamanetworkopen.2024.29702)
Supplement: Supplement 1. — Trial Protocol [file jamanetwopen-e2429702-s001.pdf]

PROTOCOL

Effect of Tourniquet on Local Tissue Concentrations of Cefazolin During Total Knee Arthroplasty: A Randomized Controlled Trial

Principal Investigator:

Adam Hart, MD, MASc, FRCSC
McGill University Health Centre –Research Institute
Montreal General Hospital
1650 Cedar Avenue, B5.159
Montreal, QC, Canada, H3G 1A4
514-934-1934 Ext: 48500

Co-Investigators:

Michael Tanzer, MDCM, FRCSC
McGill University Health Centre –Research Institute
Montreal General Hospital
1650 Cedar Avenue, B5.159
Montreal, QC, Canada, H3G 1A4

Julien Montreuil, MD, MsC
Orthopedics Resident
Department of Orthopedics Surgery
McGill University
Julien.montreuil@mail.mcgill.ca

Daina Avizonis, PhD
McGill Goodman Cancer Research Centre
1160 Pine avenue West
Montreal, QC, Canada, H3A 1A3

Karen Smith,CRA
Orthopedic Surgery Research Assistant
Department of Orthopedics Surgery
McGill University
karenwall@hotmail.com

1. BACKGROUND AND SIGNIFICANCE

In Canada, over 130 000 cases of primary joint arthroplasty are performed annually, and this number is increasing steadily with the aging population. [(1)] Projections from the United States estimate that, by 2030, more than 3.5 million primary joint arthroplasties will be performed annually. [(2)] Although rare, with reported rates of 0.5-2% within 2 years, periprosthetic joint infection (PJI) is a devastating complication with serious morbidity. [(3)] Effective use of antibiotic prophylaxis remains an important measure to prevent progression of an intraoperative contamination of the surgical site to an overt clinical infection. [(4)] It creates a hostile environment in blood and tissue inhibiting pathogens that could contaminate the wound throughout the procedure. [(5)] In order to be effective, the concentration of antibiotic must exceed the minimum inhibitory concentration (MIC) of the organism between skin incision and wound closure. [(6, 7)] *S. aureus* and Coagulase-negative staphylocci (CoNS), including *S.epidermidis*, cause close to half of deep infections and reported MIC ranges from 0.5 to 8 ug/ml in bone. [(5, 8, 9)] Achieving fourfold MIC in tissue is recommended for halting the pathogen [(10)] Cefazolin, efficient against most common pathogens in orthopaedics, has a good tissue penetration, minimal toxicity, low cost, and therefore is the antibiotic of choice in arthroplasty procedures [(3, 5)] Pharmacokinetics studies have showed that Cefazolin achieves peak bone concentrations 40 minutes after parenteral application and based on systemic dosage methods, guidelines recommend that the antibiotic should be infused within 60 minutes before surgical incision. [(3, 11-13)] Compared to conventional systemic dosing, modern techniques using liquid chromatography and mass spectrometry can adequately measure antibiotic concentration in tissue like fat and bone.[(14, 15)]

Tourniquet inflation during total knee arthroplasty (TKA) is commonly used to reduce bleeding in the surgical field; thereby facilitating exposure and cementation. However, reducing circulation to the leg may also reduce antibiotic distribution to the peri-incisional tissues. Once inflated, further parenteral addition of antibiotics is not likely to achieve peak concentration. Some studies propose techniques of regional prophylaxis with a tourniquet to achieve higher cefazolin tissue concentrations. [(8)] To our knowledge, the effect of tourniquet application on antibiotic tissue concentrations during total knee arthroplasty has not been explored. Furthermore, the effect of time

from dose to incision, patient weight, and length of surgery on local tissue concentrations of Ancef are poorly understood. Considering that infections remain the leading source of early reoperation and revision surgery, insight and optimization of local tissue antibiotics is of paramount interest.

This study looks at patients who have been referred to our clinic for a potential TKA. Once it has been determined that the patient requires a knee replacement and the patient is placed on our surgical waitlist, the patient will be asked if he or she would like to participate in the study. This randomized controlled trial would include collecting standard preoperative information, agreeing with autologous tissue collection, providing informed consent to the randomization and proceeding with standard post-operative follow-up.

2. OBJECTIVES, HYPOTHESIS AND STUDY QUESTIONS

The primary outcome of this study is to determine the local tissue concentrations of Cefazolin in serum, fat and bone during a primary TKA. The intervention in this study will be the application of a tourniquet or no tourniquet during the procedure. Secondary outcomes include: time from dose to incision, patient weight, and length of surgery on local tissue concentrations. We hypothesize that application of tourniquet will limit peak concentration of Cefazolin, while prolonging its elimination. We also think that an infusion from 10-60 minutes will be equivalent in antibiotic distribution throughout the surgery and that severe obesity significantly affects Cefazolin tissue concentration.

Risks and Benefits

This research does not involve any change in the standard of care for treating this condition. Both treatment groups are well documented, acceptable standards of clinical practice and participants will not be exposed to any additional risk other than what is routinely done during a total knee arthroplasty. This trial does not involve any new experimental treatment methods.

3. STUDY METHODS

Study Design

This will be a prospective, single centre randomized controlled trial to study patients presenting at the Montreal General Hospital for TKA. Cefazolin quantification methods will be conducted by the Goodman Cancer Research Centre Metabolomics Facility.

Study Population

Adults who require a primary total knee replacement.

Inclusion/exclusion Criteria

Inclusion Criteria:

- Adults ages 18-85 who require a primary total knee replacement
- Any gender
- Osteoarthritis, rheumatoid arthritis, avascular necrosis

Exclusion Criteria:

- Severe allergy to antibiotic used in the study
- Severe renal dysfunction (eGFR < 30 ml/min)
- MRSA colonization
- Patients who require revision surgery

Sample size

50 patients – providing approximately 25 patients in each arm of randomization. Sample size was calculated for a double-sided test with an alpha of 5%, Power of 90%, predicted difference of 25% (5 ug/g) between group and an expected standard deviation of 5 ug/g in the tissue concentration measurement based on previous studies.[7, 8, 16]

Subject recruitment, enrolment and consent process

Patients who are identified as needing a total knee replacement will be asked if they would like to participate in a study looking at antibiotic concentration throughout their surgery. If the patient agrees, the Research Assistant then will explain the study to the patients and obtain consent.

Randomization

A randomized number generator will be used to create a 1:1 allocation scheme for randomization of the patient into one of the two treatment groups. An online

randomization system will be employed to deliver the treatment group and provide a sequential study identification number. Allocations will be placed in numbered, opaque, sealed envelopes. Patients will be randomized to either the tourniquet inflation group or the no tourniquet group in the preoperative area to allow appropriate setup in the operative room.

Sample collection

Once the patient has consented to the study and has agreed to continue, standard pre-operative assessment will be conducted until the day of the surgery and randomization. The participation in this study will not affect the patients surgical date or eligibility for surgery. After the randomization described previously, the surgical team will conduct standard perioperative procedures. Patients will be given 2 g of Cefazolin (3g if >120 kg) systemically through a forearm vein. Time of infusion will be noted and a running timer will be started from that point. The infusion will need to be completed at least 10 minutes before inflation or skin incision. Time of tourniquet inflation, if applicable, and skin incision will be noted. A first sample of serum and fat will be obtained right after skin incision. An alarm will ring every 20 minutes from the time of Ancef administration on the recurring basis to obtain samples (Serum, Fat & Bone). Sample of subcutaneous fat and cancellous bone should measure at least 0.5 cm^2, using a curette in the distal femur to harvest cancellous bone. Time of definitive implants insertion will also be noted. Finally, a serum sample will be obtained one to two hours after the surgery.

After the samples have been collected, they will be labeled with a randomization number, any personal information relating to the patient will be removed. After surgery, the tissue that was collected will be sent to the Goodman Cancer Research Centre for analysis.

Sample manipulation

A pilot study will be carried by the metabolomics facility for the extraction and liquid chromatography technique on mice samples with known concentrations of cefazolin. Then, for the present study, blood and fat sample will be rinsed with normal saline solution to remove excess of blood. Sample will be stored before undergoing analysis. Blood sample will be centrifuged and prepared to adequately dose Total & Free

Cefazolin in plasma. Bone and fat samples will be finely cut with a scalpel, placed in a buffer solution, centrifuged and prepared to measured Total adipose, Interstitial adipose and bone tissue concentration.

<u>Blinding</u>

Tissue concentrations of Cefazolin being the measured outcomes, blinding will not affect validity of the results.

<u>Statistics analysis plan</u>

Means, standard deviations, and the 95% confidence intervals will be calculated for the cefazolin concentrations in the different samples. We will execute Student T-Test and Analysis of variance for repeated measures of Cefazolin concentration between groups while adjusting for by Age, BMI, length of the surgical procedure and other parameters like blood loss and fluid resuscitation. Chi-squared test for categorical data will also be performed.

<u>End Points</u>

Expected timeframe is 12-24 months for data collection, analysis and manuscript preparation

4. MANAGEMENT AND ADMINISTRATION

The Principle Investigator and Co-Investigators are responsible for the management of this study. Authorization, user access and user rights will be assigned by the Principle Investigator and Co-Investigators to their delegates. All persons working in a clinical setting and accessing patient data will be required to have RI-MUHC research privileges and will be trained in both Good Clinical Practice (GCP) and the RI-MUHC Standard Operating Procedures (SOPs)

<u>Data Collected</u>

We will collect a medical history, current and past surgical information, gender, age, BMI, diagnosis for surgery and any other information that may be pertinent. We will also collect data during the procedure: randomization group, ASA score, time of antibiotic infusion, time of tourniquet inflation and deflation, time associated will all

the tissue sampling, length of the procedure, blood loss and fluid resuscitation requirements. Post-operative data collection including adverse effect to the antibiotics, post-operative wound complication and clinical visit information will also be recorded. This information will be collected and kept in a secure double locked location. The information collected will only be accessible to research personnel authorized by the Principle Investigator and Co-Investigators.

Data Retention

Data related to the patient will be kept for up to 7 years as per REB requirements. If after 7 years, it is decided to keep the information longer, the data will be made anonymous. If after 7 years identifiable information is still required, every effort will be made to contact the patient and re-consent them.

5. CONFIDENTIALITY AND SECURITY

Only members authorized by the Principle Investigator and Co-Investigators will have access to the information. All personal and health information that is collected will remain confidential and secure to the extent permitted by applicable laws.

Adverse Events

No adverse events are expected in this study. Any adverse events will be reported as per the MUHC/Research Institute policies.

Disseminating Results

Results and findings will be communicated through group meetings, oral presentations, resident teachings and journal publications. The subjects' personal information will never be linked to any of the scientific data in question.

Withdrawal of a Participant

If a patient decides to withdraw their consent/assent, all data collected up to that point will be deleted and will not be used.

Publication

Findings from studies resulting from the information we gathered may be published: however, the participants' names will not be used in any publication. Should the

results of this study be used for one or more publications, the collaborators must acknowledge the McGill Arthroplasty Group in said publication.

Intellectual Property and Commercial Uses

Any business surrounding the intellectual property of commercial use will fall under the “McGill Patents and Intellectual Property Policy”.

REFERENCES

1. CIHI. Hip & Knee Replacements in Canada, 2017-2018: Canadian Joint Replacement Registry Annual Report CIHI. 2019(2019):1-53.

2. Kurtz S, Ong K, Lau E, Mowat F, Halpern M. Projections of Primary and Revision Hip and Knee Arthroplasty in the United States from 2005 to 2030. J Bone Jt Surg. 2007;89(4):780-5.

3. Bosco JA, Bookman J, Slover J, Edusei E, Levine B. Principles of Antibiotic Prophylaxis in Total Joint Arthroplasty: Current Concepts. The Journal of the American Academy of Orthopaedic Surgeons. 2015;23(8):e27-35.

4. Fletcher N, Sofianos D, Berkes MB, Obremskey WT. Prevention of Perioperative Infection. J Bone Jt Surg. 2007;89(7):1605-18.

5. Bicanic G, Crnogaca K, Barbaric K, Delimar D. Cefazolin should be administered maximum 30min before incision in total knee arthroplasty when tourniquet is used. Med Hypotheses. 2014;82(6):766-8.

6. Burke JF. The effective period of preventive antibiotic action in experimental incisions and dermal lesions. Surgery. 1961;50:161-8.

7. Yamada K, Matsumoto K, Tokimura F, Okazaki H, Tanaka S. Are Bone and Serum Cefazolin Concentrations Adequate for Antimicrobial Prophylaxis? Clinical Orthopaedics and Related Research®. 2011;469(12):3486-94.

8. Young SW, Zhang M, Freeman JT, Vince KG, Coleman B. Higher cefazolin concentrations with intraosseous regional prophylaxis in TKA. Clinical orthopaedics and related research. 2013;471(1):244-9.

9. Bhalodi AA, Housman ST, Shepard A, Nugent J, Nicolau DP. Tissue Pharmacokinetics of Cefazolin in Patients with Lower Limb Infections. Antimicrob Agents Ch. 2013;57(11):5679-83.

10. Quintiliani R, Nightingale C. Principles of Antibiotic Usage. Clinical orthopaedics and related research. 1984;190(&NA;):31-5.

11. Prokuski L, Clyburn TA, Evans RP, Moucha CS. Prophylactic antibiotics in orthopaedic surgery. Instr Course Lect. 2011;60:545-55.

12. Craig William A. State-of-the-Art Clinical Article: Pharmacokinetic/Pharmacodynamic Parameters: Rationale for Antibacterial Dosing of Mice and Men. Clin Infect Dis. 1998;26(1):1-10.

13. Steinberg JP, Braun BI, Hellinger WC, Kusek L, Bozikis MR, Bush AJ, et al. Timing of antimicrobial prophylaxis and the risk of surgical site infections: results from the Trial to Reduce Antimicrobial Prophylaxis Errors. Ann Surg. 2009;250(1):10-6.

14. Lillico R, Sayre CL, Sitar DS, Davies NM, Baron CM, Lakowski TM. Quantification of cefazolin in serum and adipose tissue by ultra high performance liquid chromatography-Tandem mass spectrometry (UHPLC–MS/MS): application to a pilot study of obese women undergoing cesarean delivery. J Chromatogr B. 2016;1031:94-8.

15. Zhang M, Moore GA, Chin PKL, Everts R, Begg EJ. Simultaneous Determination of Cefalexin, Cefazolin, Flucloxacillin, and Probenecid by Liquid Chromatography-Tandem Mass Spectrometry for Total and Unbound Concentrations in Human Plasma. Ther Drug Monit. 2018;40(6):682-92.

16. Thabit AK, Fatani DF, Bamakhrama MS, Barnawi OA, Basudan LO, Alhejaili SF. Antibiotic penetration into bone and joints: An updated review. Int J Infect Dis. 2019;81:128-36.
