# Supplementary material for: Tourniquet Use and Local Tissue Concentrations of Cefazolin During Total Knee Arthroplasty: A Randomized Clinical Trial
Source: JAMA Netw Open. 2024 Aug 23;7(8):e2429702. doi: 10.1001/jamanetworkopen.2024.29702 (PMC11344230; doi:10.1001/jamanetworkopen.2024.29702)
Supplement: Supplement 2. — eTable. Effect of Obesity in Fat LTC at All Time Points [file jamanetwopen-e2429702-s002.pdf]

Supplementary Online Content

Montreuil J, Tanzer M, Zhang YL, Rajda E, Avizonis D, Hart D. Tourniquet use and local tissues concentrations of cefazolin during total knee arthroplasty: a randomized clinical trial. *JAMA Netw Open*. 2024;7(8):e2429702. doi:10.1001/jamanetworkopen.2024.29702

eTable. Effect of Obesity in Fat LTC at All Time Points

This supplementary material has been provided by the authors to give readers additional information about their work.

© 2024 Montreuil J et al. JAMA Network Open.

eTable. Effect of Obesity in Fat LTC at All Time Points

	BMI < 30			BMI > 30			
**Time Point**	**Mean LTC (SD)**	**CI**	**n**	**Mean LTC (SD)**	**CI**	**n**	**P-value**
Incision							
All	8.9 (6.8)	6.4-11.5	30	7.1 (8.0)	3.4-10.7	21	0.38
TG	6.7 (3.0)	5.0-8.4	15	5.3 (2.0)	4.1-6.6	12	0.20
NTG	11.2 (8.8)	6.3-16.0	15	9.4 (12.0)	0.2-18.6	9	0.70
T1 (30 min)							
All	17.3 (8.5)	14.2-20.4	31	11.1 (6.3)	8.6-13.7	26	0.003
TG	15.6 (7.6)	11.5-19.6	16	10.6 (6.2)	6.9-14.4	13	0.07
NTG	19.2 (9.2)	14.1-24.3	15	11.6 (6.7)	7.6-15.7	13	0.02
T2 (60 min)							
All	15.8 (7.5)	12.9-18.7	28	12.0 (4.6)	10.1-13.9	24	0.03
TG	11.9 (3.2)	10.0-13.8	13	9.4 (4.4)	6.4-12.4	11	0.13
NTG	19.2 (8.6)	14.4-23.9	15	14.0 (3.5)	12.1-16.3	13	0.06
T3 (90 min)							
All	10.9 (3.0)	8.9-12.9	11	8.0 (4.9)	5.2-10.8	14	0.09
TG	10.6 (2.9)	7.6-13.6	6	6.1 (1.9)	4.1-8.0	6	0.01
NTG	11.2 (3.4)	6.9-15.4	5	9.4 (6.0)	4.4-14.4	8	0.6

LTC Blood (ug/ml)
LTC Solid tissues – Fat, Synovium, Bone (ug/g)
BMI: Body Mass Index
TG: Tourniquet Group
NTG: No-Tourniquet Group

© 2024 Montreuil J et al. JAMA Network Open.
