# Supplementary material for: Tourniquet Use and Local Tissue Concentrations of Cefazolin During Total Knee Arthroplasty: A Randomized Clinical Trial
Source: JAMA Netw Open. 2024 Aug 23;7(8):e2429702. doi: 10.1001/jamanetworkopen.2024.29702 (PMC11344230; doi:10.1001/jamanetworkopen.2024.29702)
Supplement: Supplement 3. — Data Sharing Statement [file jamanetwopen-e2429702-s003.pdf]

Data Sharing Statement

Montreuil. Tourniquet Use and Local Tissue Concentrations of Cefazolin During Total Knee Arthroplasty. *JAMA Netw Open*. Published August 23, 2024.
doi:10.1001/jamanetworkopen.2024.29702

Data
Data available: No
